# A coarse-to-fine cascade deep learning neural network for segmenting cerebral aneurysms in time-of-flight magnetic resonance angiography

**DOI:** 10.1186/s12938-022-01041-3

**Published:** 2022-09-27

**Authors:** Meng Chen, Chen Geng, Dongdong Wang, Zhiyong Zhou, Ruoyu Di, Fengmei Li, Sirong Piao, Jiajun Zhang, Yuxin Li, Yakang Dai

**Affiliations:** 1 Xuzhou Medical University, 209 Tongshan Road, Xuzhou, 221000 China; 2 Suzhou Institute of Biomedical Engineering and Technology, Chinese Academy of Sciences, 88 Keling Road, Suzhou, 215163 China; 3 Department of Radiology, Huashan Hospital, Fudan University, 12 Wulumuqi Middle Road, Shanghai, 200000 China; 4 Jinan Guoke Medical Engineering Technology Development Co., Ltd, Jinan, 250000 China; 5 Suzhou University of Science and Technology, 99 Xuefu Road, Suzhou, 215009 China

**Keywords:** Cerebral aneurysm, TOF-MRA, Deep learning, Cascade neural network

## Abstract

**Background:**

Accurate segmentation of unruptured cerebral aneurysms (UCAs) is essential to treatment planning and rupture risk assessment. Currently, three-dimensional time-of-flight magnetic resonance angiography (3D TOF-MRA) has been the most commonly used method for screening aneurysms due to its noninvasiveness. The methods based on deep learning technologies can assist radiologists in achieving accurate and reliable analysis of the size and shape of aneurysms, which may be helpful in rupture risk prediction models. However, the existing methods did not accomplish accurate segmentation of cerebral aneurysms in 3D TOF-MRA.

**Methods:**

This paper proposed a CCDU-Net for segmenting UCAs of 3D TOF-MRA images. The CCDU-Net was a cascade of a convolutional neural network for coarse segmentation and the proposed DU-Net for fine segmentation. Especially, the dual-channel inputs of DU-Net were composed of the vessel image and its contour image which can augment the vascular morphological information. Furthermore, a newly designed weighted loss function was used in the training process of DU-Net to promote the segmentation performance.

**Results:**

A total of 270 patients with UCAs were enrolled in this study. The images were divided into the training (*N* = 174), validation (*N* = 43), and testing (*N* = 53) cohorts. The CCDU-Net achieved a dice similarity coefficient (DSC) of 0.616 ± 0.167, Hausdorff distance (HD) of 5.686 ± 7.020 mm, and volumetric similarity (VS) of 0.752 ± 0.226 in the testing cohort. Compared with the existing best method, the DSC and VS increased by 18% and 5%, respectively, while the HD decreased by one-tenth.

**Conclusions:**

We proposed a CCDU-Net for segmenting UCAs in 3D TOF-MRA, and the obtained results show that the proposed method outperformed other existing methods.

## Background

Cerebral aneurysms (CAs) are abnormal bulges that mostly occur in the circle of Willis [1]. Rupture of CAs is the leading cause of subarachnoid hemorrhage (SAH) [2]. Meanwhile, the death and disability rate caused by the first rupture is as high as approximately 30% [3]. Accurate segmentation and reliable analysis of the size and shape of unruptured cerebral aneurysms (UCAs) may be helpful in rupture risk prediction [4]. At the same time, three-dimensional time-of-flight magnetic resonance angiography (3D TOF-MRA) has become one of the most commonly used screening methods because of its noninvasiveness in recent years. Hence, accurate segmentation for UCAs from 3D TOF-MRA images is particularly crucial.

However, due to the various shapes and complex locations of UCAs, the accurate segmentation of UCAs can sometimes be difficult. With the development of deep learning technologies, technical methods based on deep learning models (DLMs) would increase the speed of clinical diagnosis workflow without compromising accuracy. However, the accurate segmentation of UCAs is still challenging. According to the investigation, the study of Sichtermann et al. [5]. was the first to use a convolutional neural network (CNN) to segment UCAs on a 3D TOF-MRA data set, and the dice similarity coefficient (DSC) reached 0.53. They focused on the preprocessing method of the data set while omitting consideration of the segmentation accuracy, which resulted in a low DSC. In addition, Junma [6] at the ADAM 2020 Challenge (https://adam.isi.uu.nl/) trained nnU-Net [7] and achieved a DSC of 0.41, ranking first. They fed the entire image into the model while overlooking the problem of potential feature disappearance with the increasing depth of the model. In this paper, we developed a CCDU-Net for segmenting UCAs in 3D TOF-MRA. In detail, the CCDU-Net was based on a coarse-to-fine segmentation framework. The coarse segmentation model we used was a CNN and the fine segmentation model was the DU-Net we proposed. The dual-channel inputs of DU-Net were composed of vessel image and vascular contour image that could augment the morphological information of vessels with UCAs. Meanwhile, a weighted loss function was designed to adaptively assign weights to the voxels not well-segmented.

## Results

### Data materials

**Fig. 1:**
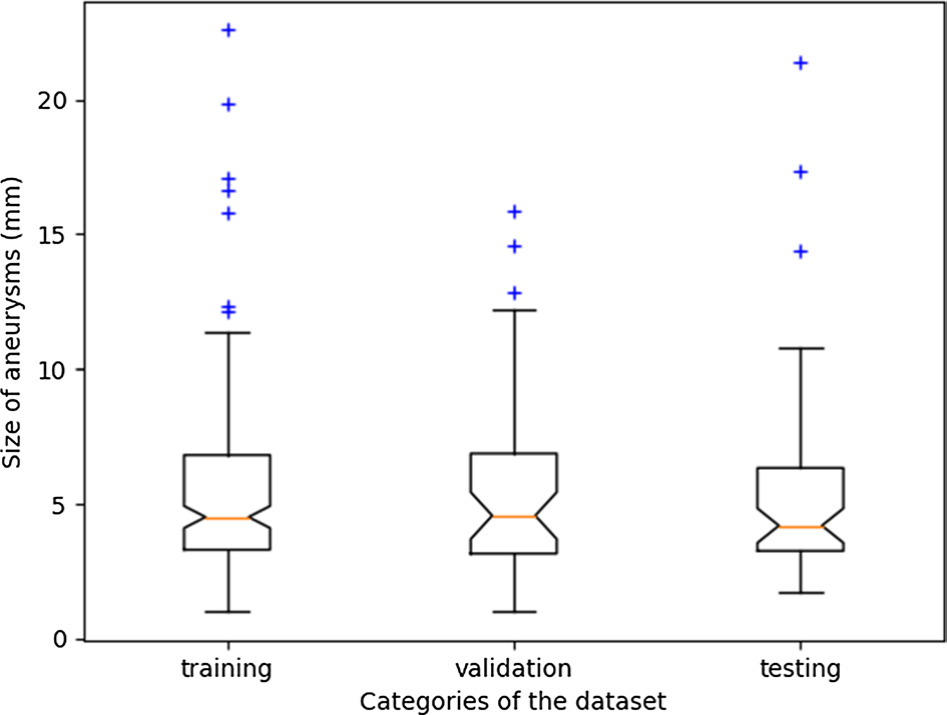
Distribution of size of aneurysms in training, validation and testing cohorts

In total, 270 patients from 2014 to 2021 were included in this retrospective study and annotated by three junior radiologists and a senior radiologist. The patients were randomly split into the training and validation cohort (*N* = 217), and the testing cohort (*N* = 53). The average size of UCAs was 5.468 ± 3.283 mm in the training and validation cohort (mean age, 61.4 ± 12.2 years), and 5.373 ± 3.515 mm in the testing cohort (mean age, 59.4 ± 13.9 years), respectively. As shown in Table 1, the distribution of aneurysms in all cohorts covered the internal carotid artery area, middle cerebral artery area, anterior cerebral artery area, posterior cerebral artery area, and basilar artery area, but no vertebral artery area was included in the testing cohort. In addition, the distribution of size of aneurysms can be seen in Fig. 1. As we adopted fivefold cross-validation strategy, we plotted a box plot of one of the folds for display.

**Table 1:** Profiles of patients

Characteristic	Training cohort + validation cohort	Testing cohort
Number of patients	217	53
Age (years)	61.4 ± 12.2	59.4 ± 13.9
Gender		
Male	89	21
Female	128	32
Size of aneurysms (mm)	5.468 ± 3.283	5.373 ± 3.515
Number of aneurysms	228	57
Location of aneurysms		
Internal carotid artery area	125	35
Middle cerebral artery area	36	7
Anterior cerebral artery area	35	9
Posterior cerebral artery area	18	3
Basilar artery area	8	3
Vertebral artery area	5	0

### CCDU-Net segmentation performance evaluation

Using our proposed method to segment UCAs in the testing cohort, CCDU-Net achieved the DSC, HD, and VS of 0.616 ± 0.167, 5.946 ± 6.680, and 0.752 ± 0.226, respectively.

The findings showed that most single UCA of 3D TOF-MRA images (36/49, segmented/total number) were well-segmented, and the distribution covered the internal carotid artery area (23/31), the middle artery area (5/6), the anterior cerebral artery area (7/8), the posterior cerebral artery area (1/1), and the basilar artery area (2/3). Double UCAs of images (2/4) were also well-segmented, and the distribution covered the posterior cerebral artery area (1/1). At the same time, double UCAs of images (2/4) that were not segmented had a distribution that covered the internal cerebral artery area (2/2) and the max diameter ranges from 2.59 to 3.08 mm.

### Comparison between CCDU-Net and other methods

The segmentation performances in the testing cohort of our proposed CCDU-Net and other existing methods are listed in Table 2, our proposed method had higher DSC, VS, and lower HD than DeepMedic and nnU-Net. Especially in HD, the segmentation performance of CCDU-Net was one-tenth of those of DeepMedic and nnU-Net. Since the other two methods showed a large number of false positive and false negative areas, the HD values were negatively affected.

**Table 2:** Segmentation performances of the proposed method compared with other existing methods in the testing cohort

Models	DSC	HD (mm)	VS
Proposed	0.616 ± 0.167	5.946 ± 6.680	0.752 ± 0.226
DeepMedic [5]	0.286 ± 0.299	61.999 ± 71.326	0.502 ± 0.303
nnU-Net [6]	0.521 ± 0.287	59.598 ± 83.901	0.717 ± 0.245

**Fig. 2:**
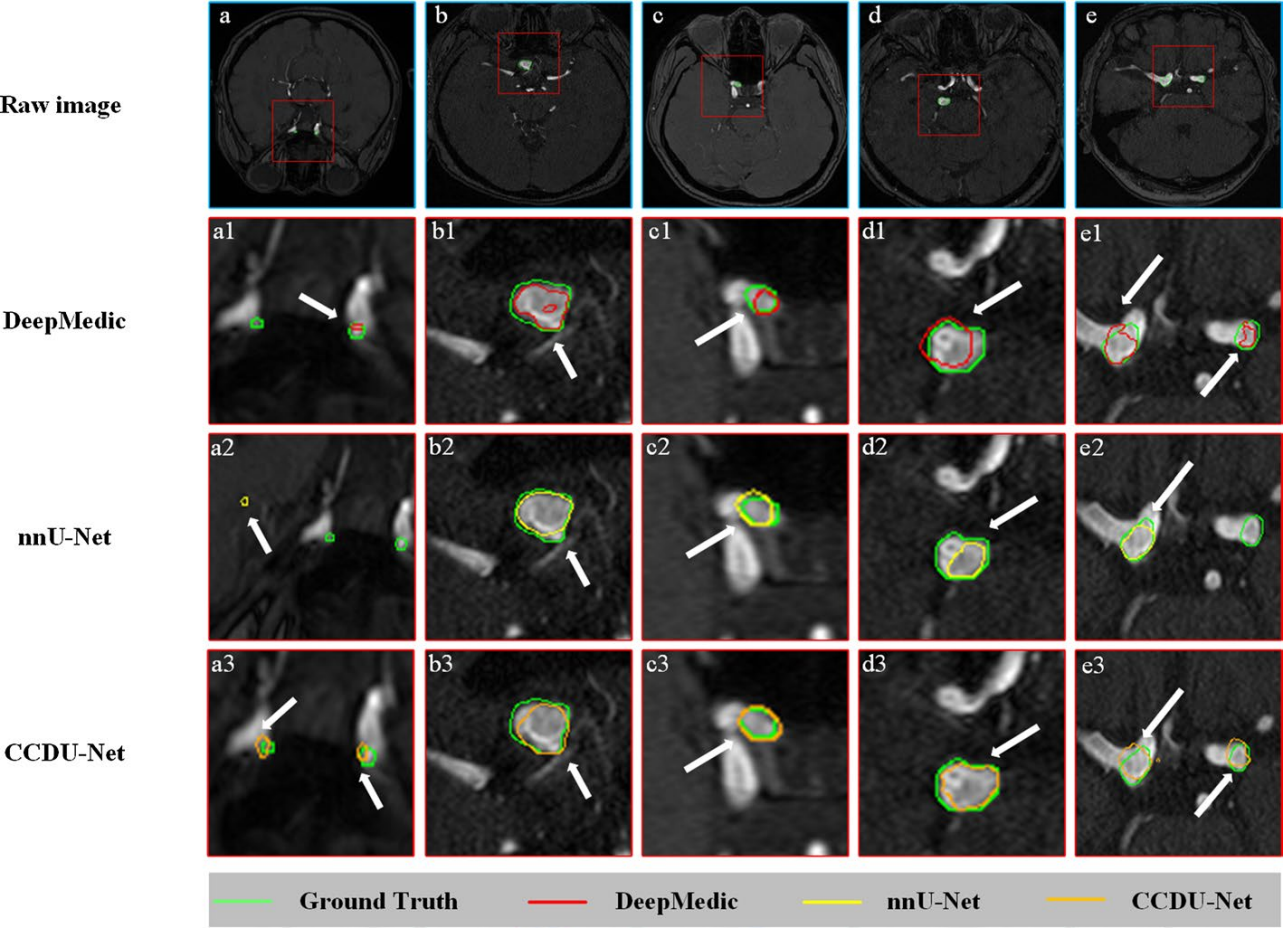
Typical visualizations of segmentation performances of different models in the testing cohort. The segmentation results of DeepMedic (red), nnU-Net (yellow), and CCDU-Net (orange) were superimposed over the GT (green). In addition, the white arrows represented the position of the segmentation results of the models

To visually display the segmentation performances of the models mentioned above, several typical TOF-MRA images were selected for comparison. The segmentation results obtained through the models are presented in Fig. 2. The segmentation results of DeepMedic (red), nnU-Net (yellow), and CCDU-Net (orange) were superimposed over the manual segmentation, namely, GT (green). The white arrows represented the position of the segmentation result of the models. The distance between lines of different colors can reflect the value of HD. We found that the segmentation results of the proposed method with low HD in b3, c3, and d3 images were closer to manual annotation, while the other methods did not precisely segment the accurate regions. Observing the results of series a and e, all three models did not segment double UCAs well. However, the number of false positive and false negative areas segmented by CCDU-Net was less than those of other existing methods.

### Ablation experiments of different inputs and loss functions

In this section, we explored the best settings of different inputs and weighted loss functions. Through ablation experiments in the testing cohort, the segmentation performances of different inputs were compared. As presented in Table 3, the dual-channel model trained with the initial loss function achieved higher DSC and VS of 0.591 ± 0.201, 0.738 ± 0.225, respectively, and lower HD of 5.686 ± 7.020 mm, while the single-channel-input network trained with initial loss function achieved DSC of 0.567 ± 0.205, HD of 6.433 ± 8.153 mm, and VS of 0.738 ± 0.217. When the vascular contour image was fed into the network as one of the dual-channel inputs, the DSC was increased by 5%, and HD was reduced by 0.747 mm.

**Table 3:** Ablation results of different inputs and weighted loss functions in the testing cohort

Input type	Loss function	DSC	HD (mm)	VS
Single-channel	Initial loss	0.567 ± 0.205	6.433 ± 8.153	0.738 ± 0.217
Dual-channel	Initial loss	**0.591±0.201**	**5.686±7.020**	**0.738±0.225**
	Initial loss	0.557 ± 0.197	9.002 ± 10.083	0.709 ± 0.225
Single-channel	WDL (*β* = 0.1)	**0.590±0.194**	**5.085±5.787**	**0.741±0.213**
	WDL (*β* = 0.2)	0.528 ± 0.164	5.585 ± 8.480	0.674 ± 0.221
	WDL (*β* = 0.3)	0.483 ± 0.162	6.194 ± 9.307	0.657 ± 0.241
	WDL (*β* = 0.4)	0.363 ± 0.149	6.910 ± 5.076	0.554 ± 0.241
	WDL (*β* = 0.5)	0.289 ± 0.113	9.052 ± 11.633	0.513 ± 0.245
	WDL (*β* = 0.6)	0.232 ± 0.111	8.269 ± 5.708	0.508 ± 0.276
	WDL (*β* = 0.7)	0.166 ± 0.101	9.453 ± 5.296	0.369 ± 0.269
	WDL (*β* = 0.8)	0.053 ± 0.164	12.659 ± 5.978	0.202 ± 0.195
	WDL (*β* = 0.9)	0.528 ± 0.164	17.377 ± 8.370	0.163 ± 0.213
	WDL (*β* = 1.0)	0.003 ± 0.009	31.892 ± 5.294	0.455 ± 0.314

Regarding the bias of single-channel input, it can be seen from Table 3 that when *β* was equal to 0.1, the model achieved the best DSC of 0.590 ± 0.194, HD of 5.085 ± 5.787 mm, and VS of 0.741 ± 0.213. Compared with the model trained with the initial loss function, the weighted dice loss function positively improved network performance.

## Discussion

In this study, we adopted and trained a CCDU-Net for segmenting UCAs in 3D TOF-MRA, and evaluated it in the testing cohort. The CCDU-Net was a cascade of a CNN and the proposed DU-Net. The following operations were included: the vascular contour was extracted along with the vessel image as the dual-channel inputs of DU-Net to augment morphological information of vessels with UCAs; a weighted loss function was designed to train DU-Net to improve the accuracy of voxels that were difficult to segment. The comparisons with the existing methods showed that our proposed method had higher DSC, VS, and lower HD than DeepMedic (0.286 ± 0.299, 61.999 ± 71.326 mm, 0.502 ± 0.303, respectively), nnU-Net (0.521 ± 0.287, 59.598 ± 83.901 mm, 0.717 ± 0.245, respectively). In particular, the segmentation performance of CCDU-Net in HD was one-tenth of those of DeepMedic and nnU-Net.

Considering the tiny UCAs in 3D TOF-MRA images, the information describing the UCAs would disappear with the increasing depth of the network. Hence, we extracted the vascular contour as one channel of input to augment the morphological information of vessels with UCAs. As can be seen from the ablation results in the testing cohort, the performance of the dual-channel network was improved compared to the single-channel backbone network. Regarding the specific performance, HD was decreased by 0.747 mm compared with the backbone model. HD is the metric to assess the maximum distance between two pointsets and is sensitive to the object contour and important for analyzing the effect of dual-channel inputs on augmenting morphological information. The improvement of HD reflected that the dual-channel inputs indeed promoted the model to learn UCAs contour which meant that morphological information could be helpful during learning.

While verifying the exponential value of the weighted loss function, we chose the value within the interval for experimental comparison, and when *β* = 0.1, the network performance improved the most. Compared with the network trained with the initial loss function, the model trained with WDL increased DSC by 6%, decreased HD by 38%, and increased VS by 5%. The voxel overlap between the ground truth and the prediction could be intuitively reflected through DSC. The improvement in DSC indicated that WDL did play a role in promoting the network to focus on the voxels of aneurysms that not well-segmented during the training process.

Meanwhile, in the coarse-to-fine segmentation framework adopted in this article, the segmentation performance at the coarse segmentation stage would affect the effect of the fine segmentation. First, in the above experiments, we found that the prediction of DeepMedic cannot guarantee that the VOI contained all cerebral aneurysms after being cropped to a size of 64 × 64 × 64, and the segmentation accuracy would be influenced. Second, if there were too many FP counts at the coarse segmentation stage, the number of cropped VOIs would also increase and even negatively cause the increasing FP counts of the overall network segmentation. Our current research aimed to improve the accuracy of the segmentation of cerebral aneurysms. In our future work, we will try to reduce the false positive count in the segmentation under high-precision segmentation performance.

## Conclusions

In this paper, we proposed a CCDU-Net for segmenting UCAs from 3D TOF-MRA images, which included two main operations: extracting the vascular contour image along with the vessel image as the dual-channel inputs of DU-Net and designing a weighted loss function for training. In the experiments, the performance of CCDU-Net has been verified in ablation studies. CCDU-Net achieved the highest DSC and extraordinary HD compared with the existing methods.

## Methods

### Data collection

The data used in this paper were acquired on 1.5 T or 3 T GE Discovery MR750 and 3 T SIEMENS Verio scanners. Details of the image acquisition parameters are presented in Table 4. The inclusion criteria were as follows: (1) patients with saccular UCAs, and (2) underwent preoperative 3D TOF-MRA. The exclusion criteria were: (1) the images contained serious artifacts, which were based on the judgment of three junior radiologists and a senior radiologist. This study was approved by the Institutional Review Boards of our center and the informed consent was waived.

**Table 4:** Image acquisition parameters

Manufacturer	Field strength (T)	TR/TE (msec)	FOV (mm)	Acquisition time	Acquisition matrix	Flip angle (°)	Thickness (mm)
SIEMENS	3	21/3.4	58–90	1 min 12 s–3 min 33 s	(256−384) × (197–331)	18	0.5–1
GE	1.5	33/6.3	75–100	1 min 22 s–3 min 4 s	(288−384) × 192/195	20	1.2–1.6
	3	25/3.4	70–94	1 min 14 s–3 min 5 s	(320−384) × 192	15	1–2.4

### Development of CCDU-Net

**Fig. 3:**
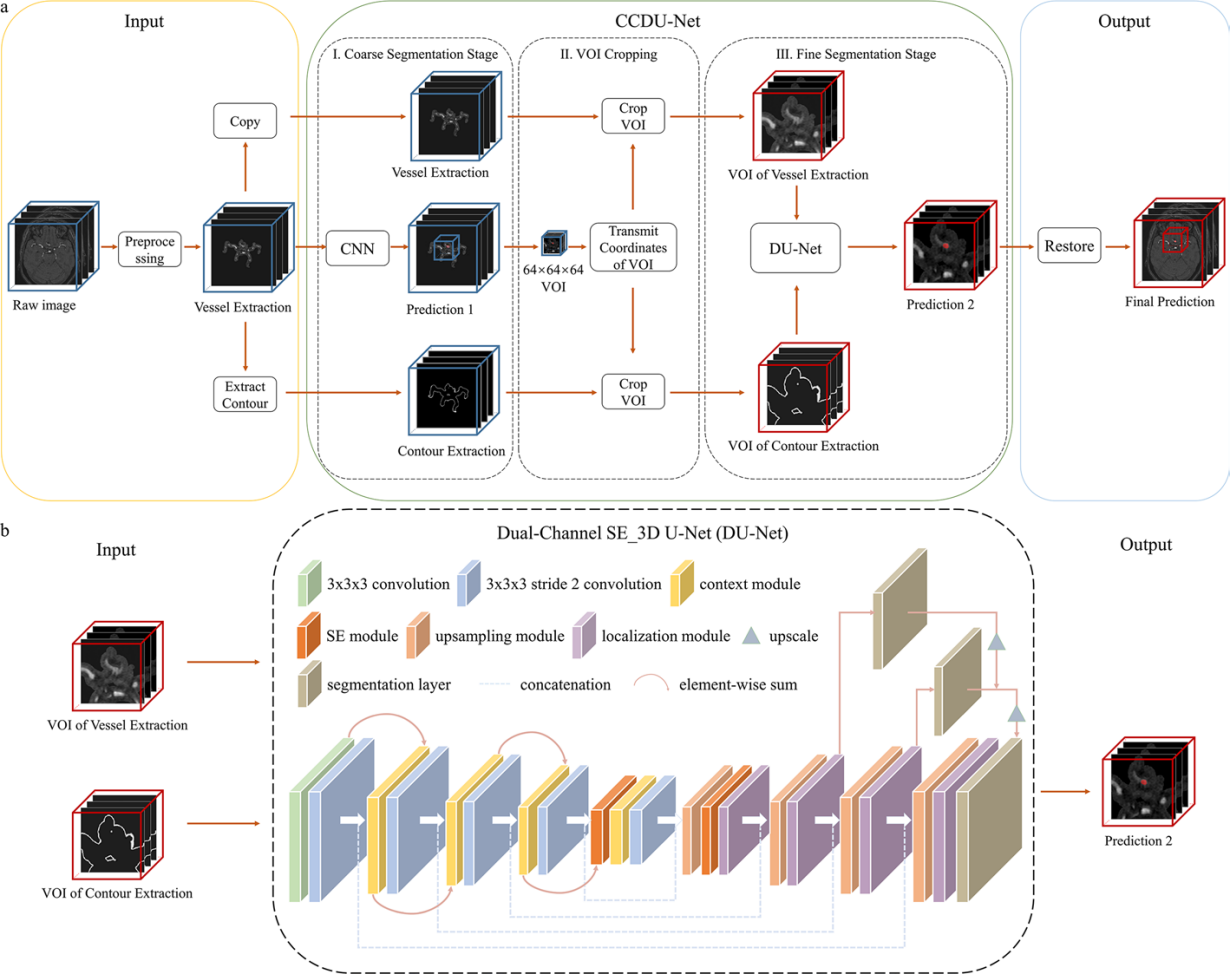
**a** Workflow of CCDU-Net we proposed. **b** Full architecture of DU-Net

In this study, we adopted CCDU-Net which was a cascade of a CNN for coarse segmentation and the DU-Net for fine segmentation, and the general workflow of CCDU-Net is shown in Fig. 3a.

First, the preprocessed data were passed through a CNN [8] for coarse segmentation to detect UCAs. Second, the volume of interest (VOI) was generated according to the coarse segmentation result. The VOI coordinates were then transmitted for cropping the vessel image and the vascular contour image at the same position.

Subsequently, VOIs of vessel image and vascular contour image were fed into DU-Net trained with our proposed weighted loss function. Figure 3b shows the architecture of DU-Net. The dual-channel inputs of DU-Net comprised VOIs of vessel image and its contour image. The network was optimized based on the variant 3D U-Net proposed by Isensee [9]. The network was still a four-layer deep structure. In the encoding path, except for the input layer, which was a 3 × 3 × 3 convolution with stride 1, each layer consisted of a 3 × 3 × 3 convolution with stride 2 followed by a context block. The context block was composed of a 3 × 3 × 3 convolution with stride 2 followed by a dropout layer with a dropout probability of 0.3. In addition, residual connections were embedded between the convolution block and the context block to reduce the loss of feature maps. After the penultimate context block, the SE [10] block was embedded. The SE block can assign weights to effective feature channels and suppress invalid features as a spatial attention mechanism. In the decoding path, with the expectation of the output layer that was 3 × 3 × 3 convolution with stride 1, the other layers consisted of a localization block and an upsampling block. After the first up-sampling block, we also embedded the SE block. The localization block included a 3 × 3 × 3 convolution with stride 1 and a 1 × 1 × 1 convolution, which input was the summation that concatenated the output of the upsampling block and the context block. Meanwhile, segmentation layers were employed for deep supervision at different layers in the decoding pathway. The final output was obtained by adding outputs of segmentation layers and activating with the SoftMax function.

Finally, the fine segmentation result was restored to the corresponding position and size of the raw image by resampling, to obtain the segmentation of the aneurysm.

### Weighted dice loss function

Inspired by the focal loss [11], we designed a new weighted dice loss function. The purpose was to promote the network to focus on the voxels of UCAs that were difficult to segment. During the training process, the overlap between label and prediction was evaluated by DSC. When the overlap was too small, the segmentation performance was poor. At this time, the loss value was weighted. That is to say, the worse the segmentation performance was, the higher the loss value would be, or vice versa. GT was the abbreviation of ground truth, Pred referred to prediction, and the symbol “||” meant absolute value. *β* and *S* were average constant terms, *β* was derived from experimental inference, and *S* was a practical value of 0.0001. The formula of our loss function is as shown in (1) below:

1 $$ {\text{Weighted Dice Loss }} = \left( {1 - {\text{DSC}}} \right)^{\beta } \left( { - 2 \times \frac{{|{\text{GT}}| \times |{\text{Pred}}| + \frac{S}{2}}}{{{\text{GT}} + {\text{Pred}} + S}}} \right) $$

To satisfy the purpose raised above, we analyzed the value interval of *β*. Since the calculation formula of DSC was similar to the above parenthetical expression, we set ${\text{Weighted loss}} = - {\text{DSC}}(1 - {\text{DSC}})^{\beta }$, and speculated that in a certain interval of *β*, when DSC was smaller, the weighted loss would increase; when DSC was larger, the weighted loss would decrease. By analyzing the monotonicity of function, it could be concluded that when the value interval of *β* was [0,1] the aim of assigning weights to poorly segmented voxels could be achieved. With the preceding analysis, we referred to the value of the exponential in focal loss function [11] and employed arithmetic progression to set the value of *β*. Models trained with different loss functions were compared based on three metrics and it can be seen from Table 3 that the best setting was *β* equalled to 0.1.

### Training of CCDU-Net

#### Preprocessing and data augmentation

The data set used in our study being from seven centers. Therefore, data preprocessing was essential to ensure feature similarity. We performed the following operations for preprocessing: (I) N4BiasFieldCorrection [12], (II) cerebral artery extraction [13], (III) Z-Score normalization [14], and (IV) vascular contour extraction. Among them, the vascular contour was extracted by the Sobel [15] operator. The processed data set was divided into the training cohort, validation cohort, and testing cohort, and the following further processing was done for the training and validation cohorts.

In the coarse-to-fine segmentation framework, the inputs were different. For the CNN, the input was a single-channel vessel image of 128 × 128 × 128. When training the CNN, we adaptively dilated the aneurysm (label = 1) according to the UCA size; For the DU-Net, the inputs were dual-channel images comprising of VOIs of vessel image and vascular contour image. In detail, we took the centroid of the label as the centre of a cube and cropped the VOIs of 64 × 64 × 64. In addition, motivated by the augmentation approaches delivered in brain-tumor segmentation [16], the training cohort was augmented eight times through flipping along the *z*-axis, discrete Gaussian filtering [17], and histogram equalization. In detail, we first performed flipping on the initial data set for augmentation twice, and then Gaussian filtering was performed on the initial data set with the flipped data set for augmentation forth. Finally, the histogram equalization was performed for eighth augmentation.

#### Training

**Fig. 4:**
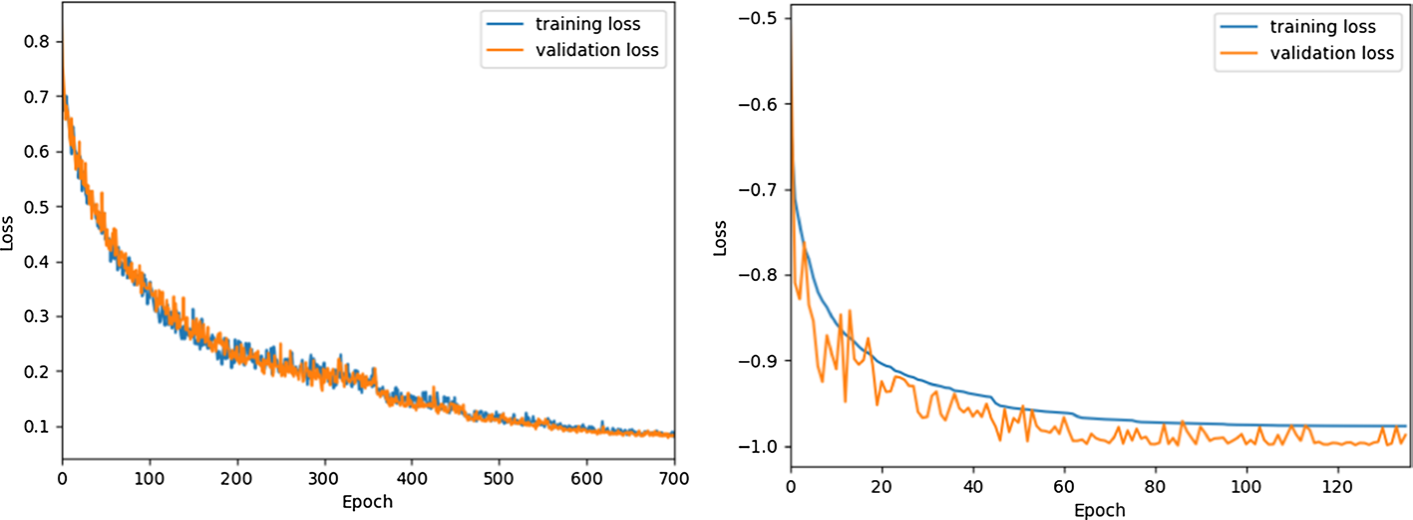
Training curves of the CNN (left) and DU-Net (right)

The training was divided into two stages: first, when training CNN, we performed fivefold cross-validation on a Tesla V100 (NIVIDA) GPU with 16-GB VRAM. The primary software environment included: Python 3.6, CUDA 10.0, and TensorFlow-GPU 1.14.0. In this article, the following parameters were set: the number of iterations was 700; the batch size was 10; the learning rate was 1e−3 initially and dropped gradually; L1 was regularized to 1e−6, L2 was regularized to 1e−4; RmsProp [18] was used as an optimizer. Second, when training DU-Net, we performed fivefold cross-validation on a GeForce RTX 2080 Ti (NIVIDA) GPU with 11-GB VRAM. The primary software environment included: Python 3.6, CUDA 10.0, Keras 2.3.1, and TensorFlow-GPU 2.0.0. In this article, the following parameters were set: the number of iterations was 500; the batch size was 1; the learning rate was 5e−4 initially and decreased to 1/2 of the last time if the validation loss did not improve within 10 iterations, training would be stopped after 50 epochs without the validation loss improving; Adam was used as an optimizer. The training curves of the CNN and DU-Net in one of the folds are shown in Fig. 4.

## Statistical analysis

The following metrics were used for evaluation: DSC, Hausdorff Distance (HD), and Volumetric Similarity (VS). GT was the abbreviation for ground truth which was based on the manual segmentation of three junior radiologists and the final check was performed by a senior radiologist with experience of 21 years. Moreover, Pred referred to prediction. The formulas of these metrics are shown in (2), (3), (4):

2 $$ {\text{DSC}} = 2 \times \frac{{{\text{GT}} \cap {\text{Pred}}}}{{{\text{GT}} \cup {\text{Pred}}}} $$

3 $$ {\text{HD = max}}\left( {{\text{h}}\left( {\text{GT, Pred}} \right){\text{, h}}\left( {\text{Pred, GT}} \right)} \right) $$

4 $$ {\text{VS}} = 1 - \frac{{\left| {{\text{GT}} - {\text{Pred}}} \right|}}{{\left| {{\text{GT}}} \right| + \left| {{\text{Pred}}} \right|}} $$

## Data Availability

Not applicable.

## Abbreviations

TOF-MRA: Time-of-flight magnetic resonance angiography
DSA: Digital subtraction angiography
CNN: Convolutional neural network
UCA: Unruptured cerebral aneurysm
VRAM: Video random access memory
GPU: Graphics processing unit
CUDA: Compute unified device architecture
MCA: Middle cerebral artery
PCA: Posterior cerebral artery
ICA: Internal carotid artery
ACA: Anterior cerebral artery
BA: Basilar artery
VA: Vertebral artery
FP: False positive
